# Supplementary material for: Predictors of Adherence to Cancer-Related mHealth Apps in Cancer Patients Undergoing Oncological or Follow-Up Treatment—A Scoping Review
Source: Int J Environ Res Public Health. 2022 Oct 21;19(20):13689. doi: 10.3390/ijerph192013689 (PMC9603736; doi:10.3390/ijerph192013689)
Supplement: Supplementary file 1 [file ijerph-19-13689-s001.zip › ijerph-1931685-supplementary.pdf]

Supplementary Material S1: Search strategy.

All terms used for the electronic database search in **PubMed:**

| No. | P | I | O | Hits |
|---|---|---|---|---|
| 1 | cancer [MeSh] OR cancer [All Fields] OR cancer* | "mobile applications" [MeSh] OR "mobile applications" [All Fields] | (adhere* [All Fields] OR adopt* [All Fields] OR accept* [All Fields] OR engage* [All Fields] OR usab* [All Fields] OR usage [All Fields] OR eval*[All Fields] OR feasab* [All Fields]) | 345 |
| 2 | cancer [MeSh] OR cancer [All Fields] OR cancer* | telemedicine[MeSH Terms] OR "telemedicine"[All Fields] | (adhere* [All Fields] OR adopt* [All Fields] OR accept* [All Fields] OR engage* [All Fields] OR usab* [All Fields] OR usage [All Fields] OR eval*[All Fields] OR feasab* [All Fields]) | 1333 |
| 3 | cancer [MeSh] OR cancer [All Fields] OR cancer* | "mhealth's"[All Fields] OR "mhealth"[All Fields] | (adhere* [All Fields] OR adopt* [All Fields] OR accept* [All Fields] OR engage* [All Fields] OR usab* [All Fields] OR usage [All Fields] OR eval*[All Fields] OR feasab* [All Fields]) | 443 |
| 4 | "radiotherapy"[MeSH Terms] OR "radiotherapy"[All Fields] OR "radiotherapies"[All Fields] | "mobile applications" [MeSh] OR "mobile applications" [All Fields] | (adhere* [All Fields] OR adopt* [All Fields] OR accept* [All Fields] OR engage* [All Fields] OR usab* [All Fields] OR usage [All Fields] OR eval*[All Fields] OR feasab* [All Fields]) | 14 |
| 5 | "radiotherapy"[MeSH Terms] OR "radiotherapy"[All Fields] OR "radiotherapies"[All Fields] | telemedicine[MeSH Terms] OR "telemedicine"[All Fields] | (adhere* [All Fields] OR adopt* [All Fields] OR accept* [All Fields] OR engage* [All Fields] OR usab* [All Fields] OR usage [All Fields] OR eval*[All Fields] OR feasab* [All Fields]) | 68 |
| 6 | "radiotherapy"[MeSH Terms] OR "radiotherapy"[All Fields] OR "radiotherapies"[All Fields] | "mhealth's"[All Fields] OR "mhealth"[All Fields] | (adhere* [All Fields] OR adopt* [All Fields] OR accept* [All Fields] OR engage* [All Fields] OR usab* [All Fields] OR usage [All Fields] OR eval*[All Fields] OR feasab* [All Fields]) | 15 |
| 7 | onco* OR "oncology"[All Fields] OR "oncology's"[All Fields] | "mobile applications" [MeSh] OR "mobile applications" [All Fields] | (adhere* [All Fields] OR adopt* [All Fields] OR accept* [All Fields] OR engage* [All Fields] OR usab* [All Fields] OR usage [All Fields] OR eval*[All Fields] OR feasab* [All Fields]) | 128 |
| 8 | onco* OR "oncology"[All Fields] OR "oncology's"[All Fields] | telemedicine[MeSH Terms] OR "telemedicine"[All Fields] | (adhere* [All Fields] OR adopt* [All Fields] OR accept* [All Fields] OR engage* [All Fields] OR usab* [All Fields] OR usage [All Fields] OR eval*[All Fields] OR feasab* [All Fields]) | 614 |
| 9 | onco* OR "oncology"[All Fields] OR "oncology's"[All Fields] | "mhealth's"[All Fields] OR "mhealth"[All Fields] | (adhere* [All Fields] OR adopt* [All Fields] OR accept* [All Fields] OR engage* [All Fields] OR usab* [All Fields] OR usage [All Fields] OR eval*[All Fields] OR feasab* [All Fields]) | 164 |
| 10 | "neoplasms"[MeSH Terms] OR "neoplasms"[All Fields] | "mobile applications" [MeSh] OR "mobile applications" [All Fields] | (adhere* [All Fields] OR adopt* [All Fields] OR accept* [All Fields] OR engage* [All Fields] OR usab* [All Fields] OR usage [All Fields] OR eval*[All Fields] OR feasab* [All Fields]) | 194 |
| 11 | "neoplasms"[MeSH Terms] OR "neoplasms"[All Fields] | telemedicine[MeSH Terms] OR "telemedicine"[All Fields] | (adhere* [All Fields] OR adopt* [All Fields] OR accept* [All Fields] OR engage* [All Fields] OR usab* [All Fields] OR usage [All Fields] OR eval*[All Fields] OR feasab* [All Fields]) | 873 |
| 12 | "neoplasms"[MeSH Terms] OR "neoplasms"[All Fields] | "mhealth's"[All Fields] OR "mhealth"[All Fields] | (adhere* [All Fields] OR adopt* [All Fields] OR accept* [All Fields] OR engage* [All Fields] OR usab* [All Fields] OR usage [All Fields] OR eval*[All Fields] OR feasab* [All Fields]) | 129 |
| | | | **Sum** | **4320** |

All terms used for the electronic database search in **CINAHL:**

| No. | P | I | O | Hits |
|---|---|---|---|---|
| 1 | ((MH "Cancer Patients") OR "cancer care" OR (MH "Cancer Survivors") OR "cancer survivors" OR cance* OR carci*) | ((MH "Mobile Applications") OR "mobile applications" OR "mobile apps" ) | (adhere* OR adopt* OR accept* OR engage* OR usab* OR usage OR eval* OR feasab* OR (MH "Motivation") OR "engagement and motivation" OR (MH "Usability Study")) | 181 |
| 2 | ((MH "Cancer Patients") OR "cancer care" OR (MH "Cancer Survivors") OR "cancer survivors" OR cance* OR carci*) | ((MH "Internet-Based Intervention") OR (MH "Telemedicine") OR (MH "Telehealth") OR "telemedicine" OR "mHealth" OR "mobile health" OR "mobile devices" OR "ehealth") | (adhere* OR adopt* OR accept* OR engage* OR usab* OR usage OR eval* OR feasab* OR (MH "Motivation") OR "engagement and motivation" OR (MH "Usability Study")) | 629 |
| 3 | ((MH "Cancer Patients") OR "cancer care" OR (MH "Cancer Survivors") OR "cancer survivors" OR cance* OR carci*) | ((MH "Smartphone") OR (MH "Cellular Phone") OR tablet) | (adhere* OR adopt* OR accept* OR engage* OR usab* OR usage OR eval* OR feasab* OR (MH "Motivation") OR "engagement and motivation" OR (MH "Usability Study")) | 426 |
| 4 | ((MH "Radiation Oncology") OR radiotherapy OR "radiation therapy") | ((MH "Mobile Applications") OR "mobile applications" OR "mobile apps" ) | (adhere* OR adopt* OR accept* OR engage* OR usab* OR usage OR eval* OR feasab* OR (MH "Motivation") OR "engagement and motivation" OR (MH "Usability Study")) | 11 |
| 5 | ((MH "Radiation Oncology") OR radiotherapy OR "radiation therapy") | ((MH "Internet-Based Intervention") OR (MH "Telemedicine") OR (MH "Telehealth") OR "telemedicine" OR "mHealth" OR "mobile health" OR "mobile devices" OR "ehealth") | (adhere* OR adopt* OR accept* OR engage* OR usab* OR usage OR eval* OR feasab* OR (MH "Motivation") OR "engagement and motivation" OR (MH "Usability Study")) | 25 |
| 6 | ((MH "Radiation Oncology") OR radiotherapy OR "radiation therapy") | ((MH "Smartphone") OR (MH "Cellular Phone") OR tablet) | (adhere* OR adopt* OR accept* OR engage* OR usab* OR usage OR eval* OR feasab* OR (MH "Motivation") OR "engagement and motivation" OR (MH "Usability Study")) | 35 |
| 7 | ((MH "Oncology") OR "oncology" OR (MH "Oncologic Care") OR (MH "Oncology Care Units") OR "oncology patients") | ((MH "Mobile Applications") OR "mobile applications" OR "mobile apps") | (adhere* OR adopt* OR accept* OR engage* OR usab* OR usage OR eval* OR feasab* OR (MH "Motivation") OR "engagement and motivation" OR (MH "Usability Study")) | 38 |
| 8 | ((MH "Oncology") OR "oncology" OR (MH "Oncologic Care") OR (MH "Oncology Care Units") OR "oncology patients") | ((MH "Internet-Based Intervention") OR (MH "Telemedicine") OR (MH "Telehealth") OR "telemedicine" OR "mHealth" OR "mobile health" OR "mobile devices" OR "ehealth") | (adhere* OR adopt* OR accept* OR engage* OR usab* OR usage OR eval* OR feasab* OR (MH "Motivation") OR "engagement and motivation" OR (MH "Usability Study")) | 155 |
| 9 | ((MH "Oncology") OR "oncology" OR (MH "Oncologic Care") OR (MH "Oncology Care Units") OR "oncology patients") | ((MH "Smartphone") OR (MH "Cellular Phone") OR tablet) | (adhere* OR adopt* OR accept* OR engage* OR usab* OR usage OR eval* OR feasab* OR (MH "Motivation") OR "engagement and motivation" OR (MH "Usability Study")) | 59 |
| 10 | ((MH "Neoplasms") OR (MH "Neoplasm Metastasis") OR neoplasm OR tumor) | ((MH "Mobile Applications") OR "mobile applications" OR "mobile apps" ) | (adhere* OR adopt* OR accept* OR engage* OR usab* OR usage OR eval* OR feasab* OR (MH "Motivation") OR "engagement and motivation" OR (MH "Usability Study")) | 138 |
| 11 | ((MH "Neoplasms") OR (MH "Neoplasm Metastasis") OR neoplasm OR tumor) | ((MH "Internet-Based Intervention") OR (MH "Telemedicine") OR (MH "Telehealth") OR "telemedicine" OR "mHealth" OR "mobile health" OR "mobile devices" OR "ehealth") | (adhere* OR adopt* OR accept* OR engage* OR usab* OR usage OR eval* OR feasab* OR (MH "Motivation") OR "engagement and motivation" OR (MH "Usability Study")) | 421 |
| 12 | ((MH "Neoplasms") OR (MH "Neoplasm Metastasis") OR neoplasm OR tumor) | ((MH "Smartphone") OR (MH "Cellular Phone") OR tablet) | (adhere* OR adopt* OR accept* OR engage* OR usab* OR usage OR eval* OR feasab* OR (MH "Motivation") OR "engagement and motivation" OR (MH "Usability Study")) | 347 |
| | | | **Sum** | **2465** |

All terms used for the electronic database search in **PsychINFO:**

| No. | P | I | O | Hits |
|---|---|---|---|---|
| 1 | ((DE "Terminal Cancer" OR (DE "Metastasis") OR "cancer patients" OR "cancer survivors" OR carc* OR cancer) | ((DE "Mobile Applications") OR "mobile app" OR "mobile health app") | ((DE "Smartphone Use") OR adhere* OR adopt* OR accept* OR engage* OR usab* OR usage OR eval* OR feasab* OR motivation) | 59 |
| 2 | ((DE "Terminal Cancer" OR (DE "Metastasis") OR "cancer patients" OR "cancer survivors" OR carc* OR cancer) | ((DE "Mobile Health") OR (DE "Telemedicine") OR mhealth OR ehealth) | ((DE "Smartphone Use") OR adhere* OR adopt* OR accept* OR engage* OR usab* OR usage OR eval* OR feasab* OR motivation) | 385 |
| 3 | ((DE "Terminal Cancer" OR (DE "Metastasis") OR "cancer patients" OR "cancer survivors" OR carc* OR cancer) | (Smartphone OR "cellular phone" OR (DE "Mobile Devices") OR (DE "Mobile Phones") OR Tablet) | ((DE "Smartphone Use") OR adhere* OR adopt* OR accept* OR engage* OR usab* OR usage OR eval* OR feasab* OR motivation) | 195 |
| 4 | (radiotherapy OR "radiation therapy") | ((DE "Mobile Applications") OR "mobile app" OR "mobile health app") | ((DE "Smartphone Use") OR adhere* OR adopt* OR accept* OR engage* OR usab* OR usage OR eval* OR feasab* OR motivation) | 1 |
| 5 | (radiotherapy OR "radiation therapy") | ((DE "Mobile Health") OR (DE "Telemedicine") OR mhealth OR ehealth) | ((DE "Smartphone Use") OR adhere* OR adopt* OR accept* OR engage* OR usab* OR usage OR eval* OR feasab* OR motivation) | 6 |
| 6 | (radiotherapy OR "radiation therapy") | (Smartphone OR "cellular phone" OR (DE "Mobile Devices") OR (DE "Mobile Phones") OR Tablet) | ((DE "Smartphone Use") OR adhere* OR adopt* OR accept* OR engage* OR usab* OR usage OR eval* OR feasab* OR motivation) | 6 |
| 7 | ((MM "Oncology") OR oncolo* OR "oncolo* care") | ((DE "Mobile Applications") OR "mobile app" OR "mobile health app") | ((DE "Smartphone Use") OR adhere* OR adopt* OR accept* OR engage* OR usab* OR usage OR eval* OR feasab* OR motivation) | 23 |
| 8 | ((MM "Oncology") OR oncolo* OR "oncolo* care") | ((DE "Mobile Health") OR (DE "Telemedicine") OR mhealth OR ehealth) | ((DE "Smartphone Use") OR adhere* OR adopt* OR accept* OR engage* OR usab* OR usage OR eval* OR feasab* OR motivation) | 151 |
| 9 | ((MM "Oncology") OR oncolo* OR "oncolo* care") | (Smartphone OR "cellular phone" OR (DE "Mobile Devices") OR (DE "Mobile Phones") OR Tablet) | ((DE "Smartphone Use") OR adhere* OR adopt* OR accept* OR engage* OR usab* OR usage OR eval* OR feasab* OR motivation) | 57 |
| 10 | ((DE "Neoplasms") OR neoplas*) | ((DE "Mobile Applications") OR "mobile app" OR "mobile health app") | ((DE "Smartphone Use") OR adhere* OR adopt* OR accept* OR engage* OR usab* OR usage OR eval* OR feasab* OR motivation) | 38 |
| 11 | ((DE "Neoplasms") OR neoplas*) | ((DE "Mobile Health") OR (DE "Telemedicine") OR mhealth OR ehealth) | ((DE "Smartphone Use") OR adhere* OR adopt* OR accept* OR engage* OR usab* OR usage OR eval* OR feasab* OR motivation) | 229 |
| 12 | ((DE "Neoplasms") OR neoplas*) | (Smartphone OR "cellular phone" OR (DE "Mobile Devices") OR (DE "Mobile Phones") OR Tablet) | ((DE "Smartphone Use") OR adhere* OR adopt* OR accept* OR engage* OR usab* OR usage OR eval* OR feasab* OR motivation) | 100 |
| | | | **Sum** | **1250** |
